# Supplementary material for: Relationship between dental experiences, oral hygiene education and self-reported oral hygiene behaviour
Source: PLoS One. 2022 Feb 24;17(2):e0264306. doi: 10.1371/journal.pone.0264306 (PMC8870456; doi:10.1371/journal.pone.0264306)
Supplement: S1 Table — Multiple answers possible. (DOCX) [file pone.0264306.s001.docx]

# Supporting Information - S1 Table

### **S1 Table. Questionnaire on toothbrushing frequency, time, duration, use of oral hygiene products, frequency and intention of dental visits, taught toothbrushing techniques taken from the German Oral Health Studies IV and V (translated into English).** Multiple answers possible.

Dear participant, the following questions concern your own oral hygiene. In the following, you will find questions about the manner, frequency and duration of your oral hygiene measures and the oral hygiene aids you use.

Q1: Have you ever been taught a toothbrushing technique?

[ ] Yes [ ] No [ ] I don’t know / don’t remember

Q2: If yes, by whom? __________

Q3: If yes, which toothbrushing technique have you been taught - Please check what is most likely to be correct.

[ ] From red to white (vertical method)

[ ] Circular (Fones method)

[ ] Jiggle

[ ] Jiggle and swipe out (modified bass method)

[ ] Horizontal

[ ] KAI

[ ] Other (please specify if known)

Q4: How often do you normally brush your teeth?

[ ] ≥ 3 x daily [ ] 2 x daily [ ] 1 x daily [ ] several times/week [ ] 1x/week [ ] less than 1x/week [ ] never

Q5: When do you brush your teeth (multiple answers possible)?

[ ] after getting up or before breakfast [ ] after breakfast [ ] after lunch

[ ] after dinner [ ] after snacks [ ] before I go to bed [ ] differs, when I come to think of it

Q6: How long do you brush your teeth on average? (Please try to estimate.)

[ ] approx. 30 sec. [ ] approx. 1 minute [ ] approx. 1.5 minutes [ ] about 2 minutes [ ] about 3 minutes [ ] longer than 3 minutes

Q7: Please check which products you often use for oral hygiene. You can add further products under "Other".

|  | never | rarely | sometimes | mostly | always |
| --- | --- | --- | --- | --- | --- |
| manual toothbrush |  |  |  |  |  |
| electric toothbrush |  |  |  |  |  |
| toothpaste |  |  |  |  |  |
| dental floss |  |  |  |  |  |
| toothpicks |  |  |  |  |  |
| interdental brush |  |  |  |  |  |
| mouth shower |  |  |  |  |  |
| mouthwash/rinsing solution |  |  |  |  |  |
| sugarless chewing gums |  |  |  |  |  |
| none |  |  |  |  |  |
| other |  |  |  |  |  |

Q8: When was the last time you visited the dentist?

[ ] Within the last 12 months [ ] Within the last 2 years [ ] Within the last 5 years [ ] More than 5 years ago [ ] I've never been to the dentist

Q9: Do you go to the dentist only when you have pain or discomfort? Or do you regularly or sometimes also go for check-ups?

[ ] I go for a check-up regularly (min. 1x per year) [ ] I also go to the check-up sometimes [ ] I only leave when I have pain or discomfort [ ] I'm not going to the dentist
