# Supplementary material for: Relationship between dental experiences, oral hygiene education and self-reported oral hygiene behaviour
Source: PLoS One. 2022 Feb 24;17(2):e0264306. doi: 10.1371/journal.pone.0264306 (PMC8870456; doi:10.1371/journal.pone.0264306)
Supplement: S2 Table — (DOCX) [file pone.0264306.s002.docx]

# Supporting Information – S2 Table

| **S2 Table. Frequency of responses in percentage given to questionnaire on toothbrushing frequency, time, duration, use of oral hygiene products, frequency and intention of dental visits, taught toothbrushing techniques taken from the German Oral Health Studies IV and V (translated into English).** | | | | | |
| --- | --- | --- | --- | --- | --- |
|  |  |  |  |  |  |
| **Q1: Have you ever been taught a toothbrushing technique?** (Answers in percentage) | | | | | |
| Yes | | | | | 84.7 |
| No | | | | | 4.1 |
| I don’t know /don’t remember | | | | | 11.2 |
|  |  |  |  |  |  |
| **Q2: If yes, by whom?** (The free-text answers given could be grouped into 3 supergroups; answers in percentage) | | | | | |
| Group prophylaxis | | | | | 38.8 |
| Individual prophylaxis | | | | | 51.8 |
| Family | | | | | 30.6 |
|  |  |  |  |  |  |
| **Q3: If yes, which toothbrushing technique have you been taught - Please check what is most likely to be correct.** (Answers in percentage) | | | | | |
| From red to white (vertical method) | | | | | 20.0 |
| Circular (Fones method) | | | | | 47.6 |
| Jiggle and swipe out (modified bass method) | | | | | 1.8 |
| Horizontal | | | | | 1.8 |
| KAI | | | | | 10.0 |
| Other (please specify if known) | | | | | 4.1 |
| no answer given | | | | | 14.7 |
|  |  |  |  |  |  |
| **Q4: How often do you normally brush your teeth?** (Answers in percentage) | | | | | |
| ≥ 3 x daily | | | | | 10.0 |
| 2 x daily | | | | | 81.8 |
| 1 x daily | | | | | 7.6 |
| several times/week | | | | | 0.6 |
| 1x/week | | | | | 0.0 |
| less than 1x/week | | | | | 0.0 |
| never | | | | | 0.0 |
|  |  |  |  |  |  |
| **Q5: When do you brush your teeth (multiple answers possible)?** (Yes-Answers in percentage) | | | | | |
| after getting up or before breakfast | | | | | 37.6 |
| after breakfast | | | | | 65.9 |
| after lunch | | | | | 8.8 |
| after dinner | | | | | 12.4 |
| after snacks | | | | | 1.8 |
| before I go to bed | | | | | 90.6 |
| differs, when I come to think of it | | | | | 7.6 |
|  |  |  |  |  |  |
| **Q6: How long do you brush your teeth on average? (Please try to estimate.)** (Answers in percentage) | | | | | |
| approx. 30 sec. | | | | | 0.0 |
| approx. 1 minute | | | | | 9.4 |
| approx. 1.5 minutes | | | | | 11.8 |
| about 2 minutes | | | | | 48.2 |
| about 3 minutes | | | | | 27.6 |
| longer than 3 minutes | | | | | 2.9 |
|  |  |  |  |  |  |
| **Q7: Please check which products you often use for oral hygiene! You can add further products under "Other".** (Answers in percentage) | | | | | |
|  | never | rarely | sometimes | mostly | always |
| manual toothbrush | 0.0 | 0.0 | 0.0 | 12.9 | 87.1 |
| electric toothbrush | 77.1 | 17.1 | 5.9 | 0.0 | 0.0 |
| toothpaste | 0.0 | 1.2 | 0.0 | 0.0 | 98.8 |
| dental floss | 19.4 | 30.6 | 40.0 | 8.8 | 1.2 |
| toothpicks | 60.0 | 31.8 | 8.2 | 0.0 | 0.0 |
| interdental brush | 74.1 | 15.3 | 8.2 | 1.8 | 0.6 |
| mouth shower | 95.9 | 2.9 | 1.2 | 0.0 | 0.0 |
| mouthwash/rinsing solution | 44.7 | 21.2 | 21.8 | 10.0 | 2.4 |
| sugarless chewing gums | 24.7 | 21.8 | 30.6 | 15.3 | 7.6 |
| none | 99.4 | 0.6 | 0.0 | 0.0 | 0.0 |
| other | 0.0 | 0.0 | 0.0 | 0.0 | 0.0 |
|  |  |  |  |  |  |
| **Q8: When was the last time you visited the dentist?** (Answers in percentage) | | | | | |
| Within the last 12 months | | | | | 75.3 |
| Within the last 2 years | | | | | 17.1 |
| Within the last 5 years | | | | | 5.9 |
| More than 5 years ago | | | | | 0.0 |
| I've never been to the dentist | | | | | 0.6 |
| no answer given | | | | | 1.2 |
|  |  |  |  |  |  |
| **Q9: Do you go to the dentist only when you have pain or discomfort? Or do you regularly or sometimes also go for check-ups?** (Answers in percentage) | | | | | |
| I go for a check-up regularly (min. 1x per year) | | | | | 61.4 |
| I also go to the check-up sometimes | | | | | 24.7 |
| I only leave when I have pain or discomfort | | | | | 8.2 |
| I'm not going to the dentist | | | | | 0.6 |
| no answer given | | | | | 2.4 |
