# Supplementary material for: Relationship between dental experiences, oral hygiene education and self-reported oral hygiene behaviour
Source: PLoS One. 2022 Feb 24;17(2):e0264306. doi: 10.1371/journal.pone.0264306 (PMC8870456; doi:10.1371/journal.pone.0264306)
Supplement: S3 Table — (DOCX) [file pone.0264306.s003.docx]

# Supporting Information – S3 Table

### **S3 Table. Newly composed questionnaire (translated into English).**

|  | Dear participant, the following questions deal with your feelings during oral hygiene, your motivation to brush your teeth and your satisfaction with your teeth. Please evaluate how accurate fit the following statements are for you. | | | | | | | | | | |
| --- | --- | --- | --- | --- | --- | --- | --- | --- | --- | --- | --- |
|  |  | | |  |  | |  | |  | |  |
| *D1: Feelings related to oral hygiene:* | | | | | | | | | | | |
| Itemno. | The statement is ... true. | not | barely | | | in part | | largely | | entirely | |
| 1 | I have a good feeling after cleaning |  |  | | |  | |  | |  | |
| 2 | Brushing my teeth releases a liberating feeling in me. |  |  | | |  | |  | |  | |
| 3 | I look forward to brushing my teeth. |  |  | | |  | |  | |  | |
| 4 | Brushing my teeth matters to me. |  |  | | |  | |  | |  | |
| 5 | Toothbrushing is boring. |  |  | | |  | |  | |  | |
| 6 | I do something else while brushing my teeth. |  |  | | |  | |  | |  | |
| 7 | Brushing my teeth is an annoying duty for me. |  |  | | |  | |  | |  | |
|  |  |  |  | | |  | |  | |  | |
| *D2: My motivation to brush my teeth:* | | | | | | | | | | | |
|  | The statement is true. | not | barely | | | in part | | largely | | entirely | |
| 8 | Teeth have always been important to me. |  |  | | |  | |  | |  | |
| 9 | I brush my teeth so that they look beautiful. |  |  | | |  | |  | |  | |
| 10 | I brush my teeth to keep them healthy/to not get any tooth decay. |  |  | | |  | |  | |  | |
| 11 | I brush my teeth, so that my dentist doesn't find anything on my next visit. |  |  | | |  | |  | |  | |
| 12 | I brush my teeth because one should do it just like that (out of a sense of duty). |  |  | | |  | |  | |  | |
| 13 | I clean my teeth so that the mouth gets cleaner. |  |  | | |  | |  | |  | |
| 14 | I brush my teeth so that I don't get any bad breath. |  |  | | |  | |  | |  | |
| 15 | My dentist has motivated me to brush my teeth. |  |  | | |  | |  | |  | |
| 16 | I can do a lot myself to keep my teeth healthy. |  |  | | |  | |  | |  | |
| 17 | For me everything’s too late, so brushing my teeth doesn't help me anymore. |  |  | | |  | |  | |  | |
| 18 | It’s not necessary to overdo oral hygiene. |  |  | | |  | |  | |  | |
| 19 | Everyone in my family has good teeth, so I don't worry about brushing my teeth. |  |  | | |  | |  | |  | |
| 20 | Well-groomed teeth a part of a well-groomed appearance. |  |  | | |  | |  | |  | |
|  |  |  |  | | |  | |  | |  | |
| *D3: Feeling pleasure and satisfaction:* | | | | | | | | | | | |
|  | The statement is ... true. | not | barely | | | in part | | largely | | entirely | |
| 21 | I am satisfied with my teeth. |  |  | | |  | |  | |  | |
| 22 | My teeth feel good. |  |  | | |  | |  | |  | |
| 23 | I regularly check my teeth in the mirror. |  |  | | |  | |  | |  | |
| 24 | I can rely on my teeth. |  |  | | |  | |  | |  | |
| 25 | I immediately notice any changes in my teeth. |  |  | | |  | |  | |  | |
| 26 | When I notice a dark spot on my teeth, I immediately make a dental appointment. |  |  | | |  | |  | |  | |
| 27 | I regularly feel with my tongue if my teeth are fine. |  |  | | |  | |  | |  | |
| 28 | I'm worried about having bad breath. |  |  | | |  | |  | |  | |
| 29 | I don't care whether I have a tooth gap or not. |  |  | | |  | |  | |  | |
| 30 | I've been approached about my bad breath several times. |  |  | | |  | |  | |  | |
| 31 | My partnership suffers under my teeth. |  |  | | |  | |  | |  | |
| 32 | I'm worried about being rejected because of my teeth. |  |  | | |  | |  | |  | |
| 33 | I'm ashamed of my teeth. |  |  | | |  | |  | |  | |
|  |  |  |  | | |  | |  | |  | |
|  |  |  |  | | |  | |  | |  | |
|  | Dear participant, the following questions deal with your experiences with the dentist. Please evaluate how accurate the following statements are regarding your previous visits to the dentist. | | | | | | | | | | |
|  |  | | |  |  | |  | |  | |  |
| *D4: Experiences and feelings at the dentist:* | | | | | | | | | | | |
|  | The statement is ... true. | not | barely | | | in part | | largely | | entirely | |
| 34 | I like going to the dentist |  |  | | |  | |  | |  | |
| 35 | I've always been at dental check-ups, nothing's ever had to be done. |  |  | | |  | |  | |  | |
| 36 | I find the visit to the dentist unpleasant but useful. |  |  | | |  | |  | |  | |
| 37 | Even the professional cleaning is unpleasant. |  |  | | |  | |  | |  | |
| 38 | My experiences so far have been bad. |  |  | | |  | |  | |  | |
| 39 | Just the thought of the dentist makes me feel bad. |  |  | | |  | |  | |  | |
|  |  |  |  | | |  | |  | |  | |
| *D5: Parent/Childhood/Education:* | | | | | | | | | | | |
|  | The statement is ... true. | not | barely | | | in part | | largely | | entirely | |
| 40 | My parents have taken care of my oral hygiene. |  |  | | |  | |  | |  | |
| 41 | My parents motivated my oral hygiene. |  |  | | |  | |  | |  | |
| 42 | My parents checked my toothbrushing. |  |  | | |  | |  | |  | |
| 43 | My parents regularly went to the dentist themselves. |  |  | | |  | |  | |  | |
| 44 | Brushing my teeth was a natural part of my daily routine from an early age on. |  |  | | |  | |  | |  | |
| 45 | I used to be threatened with a visit to the dentist as a punishment. |  |  | | |  | |  | |  | |
| 46 | My parents transferred their fear of dentists onto me. |  |  | | |  | |  | |  | |
| 47 | When I was a child or teenager, I often had to go see an orthodontist. |  |  | | |  | |  | |  | |
| 48 | Already early (until puberty) I had a lot of work on my teeth - I always needed new fillings. |  |  | | |  | |  | |  | |
| 49 | As a child, I felt frightened visiting the dentist. |  |  | | |  | |  | |  | |
| 50 | When I was little, I experienced dental treatment as painful. |  |  | | |  | |  | |  | |
|  |  |  |  | | |  | |  | |  | |
