# Supplementary material for: Relationship between dental experiences, oral hygiene education and self-reported oral hygiene behaviour
Source: PLoS One. 2022 Feb 24;17(2):e0264306. doi: 10.1371/journal.pone.0264306 (PMC8870456; doi:10.1371/journal.pone.0264306)
Supplement: S4 Table — Items with grey background displayed too poor communalities or were not found in the chosen six-factorial solution and were not included in the further analyses; communalities in bold letters indicate that they belong to the respective factor. (DOCX) [file pone.0264306.s004.docx]

# Supporting Information – S4 Table

**S4 Table. Rotated factor loads and communalities of the items of the newly developed questionnaires. Items with grey background displayed too poor communalities or were not found in the chosen six-factorial solution and were not included in the further analyses; communalities in bold letters indicate that they belong to the respective factor.**

|  | **Questionnaire** | **Communalities** | | | | | |
| --- | --- | --- | --- | --- | --- | --- | --- |
|  | Themes and included items | Factor “attitude towards oral hygiene” | Factor “attitude towards one’s own teeth” | Factor “sense of care“ | Factor “self-control of one’s own teeth” | Factor “ sense of obligation“ | Factor “experiences of bad breath“ |
| D1 | *Feelings related to oral hygiene:* | | | | | | |
| 1 | I have a good feeling after cleaning | **0.518** |  |  |  |  |  |
| 2 | Brushing my teeth releases a liberating feeling in me. | **0.588** |  |  | 0.394 |  |  |
| 3 | I look forward to brushing my teeth. | **0.773** |  |  |  |  |  |
| 4 | I don't really care about brushing my teeth. | -0.332 |  | -0.336 |  |  |  |
| 5 | Toothbrushing is boring. | **-0.705** |  |  |  |  |  |
| 6 | I do something else while brushing my teeth. |  |  |  |  |  |  |
| 7 | Brushing my teeth is an annoying duty for me. | **-0.689** |  |  |  |  |  |
|  |  |  |  |  |  |  |  |
|  | Themes and included items | Factor “attitude towards oral hygiene” | Factor “attitude towards one’s own teeth” | Factor “sense of care“ | Factor “self-control of one’s own teeth” | Factor “ sense of obligation“ | Factor “experiences of bad breath“ |
| D2 | *My motivation to brush my teeth:* | | | | | | |
| 8 | Teeth have always been important to me. |  |  |  |  | **0.442** |  |
| 9 | I brush my teeth so that they look beautiful. |  |  | 0.330 |  | 0.345 |  |
| 10 | I brush my teeth to keep them healthy/to not get any tooth decay. |  |  | **0.301** |  |  |  |
| 11 | I brush my teeth, so that my dentist doesn't find anything on my next visit. |  |  |  |  | **0.424** |  |
| 12 | I brush my teeth because one should do it just like that (out of a sense of duty). |  |  |  |  | **0.302** |  |
| 13 | I clean my teeth so that the mouth gets cleaner. |  |  | **0.559** |  |  |  |
| 14 | I brush my teeth so that I don't get any bad breath. |  |  | **0.763** |  |  |  |
| 15 | My dentist has motivated me to brush my teeth. |  |  |  | 0.318 |  |  |
| 16 | I can do a lot myself to keep my teeth healthy. |  |  |  | 0.352 |  |  |
| 17 | For me everything is too late, so brushing my teeth doesn't help me anymore. |  | 0.347 |  |  |  |  |
| 18 | It’s not necessary to overdo oral hygiene. |  |  |  |  |  |  |
| 19 | Everyone in my family has good teeth, so I don't worry about brushing my teeth. |  |  |  |  |  |  |
| 20 | Well-groomed teeth a part of a well-groomed appearance. |  |  | **0.552** |  |  |  |
|  |  |  |  |  |  |  |  |
|  | Themes and included items | Factor “attitude towards oral hygiene” | Factor “attitude towards one’s own teeth” | Factor “sense of care“ | Factor “self-control of one’s own teeth” | Factor “ sense of obligation“ | Factor “experiences of bad breath“ |
| *D3* | *Feeling pleasure and satisfaction:* | | | | | | |
| 21 | I am satisfied with my teeth. |  | **-0.640** |  |  |  |  |
| 22 | My teeth feel good. | 0.335 | **-0.570** |  |  | 0.355 |  |
| 23 | I regularly check my teeth in the mirror. |  |  | 0.284 | 0.295 |  |  |
| 24 | I can rely on my teeth. |  | **-0.366** |  |  |  |  |
| 25 | I immediately notice any changes in my teeth. |  |  |  | **0.459** | 0.320 |  |
| 26 | When I notice a dark spot on my teeth, I immediately make a dental appointment. |  |  |  | **0.474** |  |  |
| 27 | I regularly feel with my tongue if my teeth are fine. |  |  |  | **0.577** |  |  |
| 28 | I'm worried about having bad breath. |  |  | 0.362 |  |  | **0.557** |
| 29 | I don't care whether I have a tooth gap or not. |  |  |  |  |  |  |
| 30 | I've been approached about my bad breath several times. |  |  |  |  |  | **0.614** |
| 31 | My partnership suffers under my teeth. |  |  | -0.343 |  |  | **0.373** |
| 32 | I'm worried about being rejected because of my teeth. |  | **0.712** |  |  |  |  |
| 33 | I'm ashamed of my teeth. |  | **0.827** |  |  |  |  |
