# Supplementary material for: Relationship between dental experiences, oral hygiene education and self-reported oral hygiene behaviour
Source: PLoS One. 2022 Feb 24;17(2):e0264306. doi: 10.1371/journal.pone.0264306 (PMC8870456; doi:10.1371/journal.pone.0264306)
Supplement: S5 Table — Percentage data of the frequency of answering the items with the answer options “not”, “barely”, “in part”, “largely” or “entirely”. (DOCX) [file pone.0264306.s005.docx]

# Supporting Information – S5 Table

**S5 Table.** **Percentage of responses to the newly developed questionnaires.**

**Percentage data of the frequency of answering the items with the answer options “not”, “barely”, “in part”, “largely” or “entirely”.**

| *D1: Feelings related to oral hygiene:* | | | | | | |
| --- | --- | --- | --- | --- | --- | --- |
|  | The statement is ... true. | not | barely | in part | largely | entirely |
| 1 | I have a good feeling after cleaning | 0 | 0.6 | 4.7 | 35.3 | 59.4 |
| 2 | Brushing my teeth releases a liberating feeling in me. | 5.3 | 12.9 | 18.8 | 38.8 | 24.1 |
| 3 | I look forward to brushing my teeth. | 12.9 | 23.5 | 35.9 | 18.8 | 8.8 |
| 4 | I don’t really care about brushing my teeth. | 68.2 | 18.2 | 7.6 | 5.3 | 0.6 |
| 5 | Toothbrushing is boring. | 11.2 | 18.8 | 31.8 | 25.9 | 12.4 |
| 6 | I do something else while brushing my teeth. | 21.8 | 22.4 | 35.3 | 16.5 | 4.1 |
| 7 | Brushing my teeth is an annoying duty for me. | 0.6 | 6.5 | 25.3 | 42.9 | 24.7 |
|  |  |  |  |  |  |  |
| *D2: My motivation to brush my teeth:* | | | | | | |
|  | The statement is true. | not | barely | in part | Largely | entirely |
| *8* | Teeth have always been important to me. | 0.6 | 6.5 | 21.8 | 41.8 | 29.4 |
| 9 | I brush my teeth so that they look beautiful. | 0 | 0.6 | 2.4 | 17.1 | 80 |
| 10 | I brush my teeth to keep them healthy/to not get any tooth decay. | 0 | 0.6 | 2.4 | 17.1 | 80 |
| 11 | I brush my teeth, so that my dentist doesn't find anything on my next visit. | 14.7 | 22.4 | 27.6 | 21.2 | 14.1 |
| 12 | I brush my teeth because one should do it just like that (out of a sense of duty). | 23.5 | 31.8 | 23.5 | 15.3 | 5.9 |
| 13 | I clean my teeth so that the mouth gets cleaner. | 0.6 | 5.3 | 12.4 | 31.8 | 50 |
| 14 | I brush my teeth so that I don't get any bad breath. | 1.2 | 1.8 | 12.4 | 29.4 | 55.3 |
| 15 | My dentist has motivated me to brush my teeth. | 13.5 | 31.8 | 28.8 | 15.3 | 10.6 |
| 16 | I can do a lot myself to keep my teeth healthy. | 0 | 1.8 | 7.1 | 33.5 | 57.6 |
| 17 | For me everything’s too late, so brushing my teeth doesn't help me anymore. | 94.7 | 3.5 | 1.8 | 0 | 0 |
| 18 | It’s not necessary to overdo oral hygiene. | 30.2 | 32.5 | 29 | 6.5 | 1.8 |
| 19 | Everyone in my family has good teeth, so I don't worry about brushing my teeth. | 48.2 | 27.6 | 20 | 2.4 | 1.8 |
| 20 | Well-groomed teeth a part of a well-groomed appearance. | 0 | 0.6 | 7.6 | 22.9 | 68.8 |
|  |  |  |  |  |  |  |
| *D3: Feeling pleasure and satisfaction:* | | | | | | |
|  | The statement is ... true. | not | barely | in part | largely | entirely |
| 21 | I am satisfied with my teeth. | 1.8 | 2.9 | 20.6 | 54.7 | 20 |
| 22 | My teeth feel good. | 1.8 | 0.6 | 13.5 | 57.1 | 27.1 |
| 23 | I regularly check my teeth in the mirror. | 1.2 | 18.8 | 24.7 | 30.6 | 24.7 |
| 24 | I can rely on my teeth. | 1.8 | 4.7 | 16.5 | 42.9 | 34.1 |
| 25 | I immediately notice any changes in my teeth. | 2.9 | 11.2 | 23.5 | 35.9 | 26.5 |
| 26 | When I notice a dark spot on my teeth, I immediately make a dental appointment. | 10.6 | 19.4 | 21.8 | 20 | 28.2 |
| 27 | I regularly feel with my tongue if my teeth are fine. | 4.7 | 17.6 | 21.2 | 25.9 | 30.6 |
| 28 | I'm worried about having bad breath. | 7.6 | 19.4 | 30 | 23.5 | 19.4 |
| 29 | I don't care whether I have a tooth gap or not. | 66.5 | 20 | 8.2 | 4.2 | 1.2 |
| 30 | I've been approached about my bad breath several times. | 80 | 15.3 | 4.1 | 0.6 | 0 |
| 31 | My partnership suffers under my teeth. | 94.7 | 4.7 | 0.6 | 0 | 0 |
| 32 | I'm worried about being rejected because of my teeth. | 74.7 | 20 | 4.1 | 1.2 | 0 |
| 33 | I'm ashamed of my teeth. | 81.1 | 13 | 5.3 | 0 | 0.6 |
|  |  |  |  |  |  |  |
| *D4: Experiences and feelings at the dentist:* | | | | | | |
|  | The statement is ... true. | not | barely | in part | largely | entirely |
| 34 | I like going to the dentist | 10 | 19.4 | 27.1 | 27.1 | 16.5 |
| 35 | I've always been at dental check-ups, nothing's ever had to be done. | 50.5 | 10.6 | 12.4 | 15.3 | 11.2 |
| 36 | I find the visit to the dentist unpleasant but useful. | 14.1 | 28.2 | 17.1 | 22.9 | 17.6 |
| 37 | Even the professional cleaning is unpleasant. | 41.8 | 27.1 | 20.6 | 9.4 | 1.2 |
| 38 | My experiences so far have been bad. | 58.2 | 24.1 | 14.7 | 2.4 | 0.6 |
| 39 | Just the thought of the dentist makes me feel bad. | 72.9 | 14.7 | 7.6 | 4.1 | 0.6 |
|  |  |  |  |  |  |  |
| *D5: Parent/Childhood/Education:* | | | | | | |
|  | The statement is ... true. | not | barely | in part | largely | entirely |
| 40 | My parents have taken care of my oral hygiene. | 1.2 | 2.9 | 7.6 | 32.4 | 55.9 |
| 41 | My parents motivated my oral hygiene. | 2.4 | 3.5 | 12.9 | 31.8 | 49.4 |
| 42 | My parents checked my toothbrushing. | 6.4 | 10 | 27.1 | 17.6 | 38.8 |
| 43 | My parents regularly went to the dentist themselves. | 4.7 | 5.3 | 15.9 | 22.9 | 51.2 |
| 44 | Brushing my teeth was a natural part of my daily routine from an early age on. | 1.8 | 4.1 | 7.6 | 12.9 | 73.5 |
| 45 | I used to be threatened with a visit to the dentist as a punishment. | 95.3 | 3.5 | 1.2 | 0 | 0 |
| 46 | My parents transferred their fear of dentists onto me. | 91.8 | 5.9 | 2.4 | 0 | 0 |
| 47 | When I was a child or teenager, I often had to go see an orthodontist. | 32.9 | 8.2 | 10 | 11.8 | 37.1 |
| 48 | Already early (until puberty) I had a lot of work on my teeth - I always needed new fillings. | 62.9 | 15.3 | 11.8 | 5.3 | 4.7 |
| 49 | As a child, I felt frightened visiting the dentist. | 54.7 | 20.6 | 10.6 | 8.2 | 5.9 |
| 50 | When I was little, I experienced dental treatment as painful. | 54.7 | 19.4 | 15.9 | 6.5 | 3.5 |
