# Supplementary material for: Relationship between dental experiences, oral hygiene education and self-reported oral hygiene behaviour
Source: PLoS One. 2022 Feb 24;17(2):e0264306. doi: 10.1371/journal.pone.0264306 (PMC8870456; doi:10.1371/journal.pone.0264306)
Supplement: S1 Protocol — (PDF) [file pone.0264306.s007.pdf]

Association between personality traits, body image and brushing performance – a video observation and questionnaire based study – APBB1

Study Protocol

Prof. Dr. Almut Zeeck, PD Dr. Armin Hartmann (Klinik f. Psychosomatische Medizin u. Psychotherapie, Albert-Ludwigs-Universität Freiburg)

Prof. Dr. Nadine Schlüter (Department f. Zahn-, Mund- u. Kieferheilkunde, Klinik f. Zahnerhaltungskunde und Parodontologie, Stiftungsprofessur für Kariesforschung, Albert-Ludwigs-Universität Freiburg)

January 2017

Table of contents

1 GLOSSARY / LIST OF ABBREVIATIONS 3

2 SYNOPSIS 3

3 ADRESSES AND RESPONSIBILITIES 6
3.1 ADDRESSES 6
3.2 RESPONSIBILITIES 7

4 INTRODUCTION 7

5 STUDY OBJECTIVE 9

6 INVESTIGATIONAL PLAN 9
6.1 OVERALL STUDY DESIGN AND PLAN 9
6.2 DISCUSSION OF THE STUDY DESIGN 9
6.3 STUDY CENTRES AND TECHNICAL REQUIREMENTS 10
6.4 STUDY POPULATION 10
6.4.1 Inclusion Criteria 10
6.4.2 Exclusion Criteria 11
6.4.3 Blinding 11

7 FLOW CHART 11

8 STUDY PARAMETERS 12

9 PROCEDURES 13

10 QUALITY CONTROL AND DATA MANGEMENT 13
10.1 GOOD CLINICAL PRACTICE 13
10.2 CALIBRATION AND TRAINING OF INVESTIGATORS 14
10.3 CASE REPORT FORMS (CRF) 14
10.4 AUDITS AND INSPECTIONS 14
10.5 DATA MANAGEMENT 14

11 STATISTICAL METHODS AND DETERMINATION OF SAMPLE SIZE 15
11.1 STATISTICAL ANALYSIS PLAN 15
11.2 SAMPLE SIZE CALCULATION 16

12 PROTOCOL AMENDMENTS 16

13 ETHICS AND REGULATIONS 16
13.1 ETHICAL CONDUCT OF THE STUDY AND INDEPENDENT ETHICS COMMISSION (IEC) AND RELEVANT AUTHORITIES 16
13.2 VOLUNTEER INFORMATION AND INFORMED CONSENT 17
13.3 VOLUNTEER INSURANCE AND ALLOWANCE 17
13.4 VOLUNTEER DATA PROTECTION AND RETENTION OF STUDY RECORDS 17
13.5 PUBLICATION / PRESENTATION 17

14 SIGNATURES AND AGREEMENT WITH THE PROTOCOL 20

15 LIST OF APPENDICES 21
15.1 CRF 21
15.2 VOLUNTEERS INFORMATION AND INFORMED CONSENT 21

1 GLOSSARY / LIST OF ABBREVIATIONS

IEC	Independent Ethics Commission
CRF	Case Report Form
GCP	Good Clinical Practice
ICF	Informed Consent Form
PI	Principle Investigators
CI	Investigators

2 SYNOPSIS

**TITLE OF TRIAL**	Association between personality traits, body experience and brushing performance - a video observation and questionnaire based study
**SHORT TITLE**	Oral hygiene habits and personality structure in adults
**EUDRACT NO**	Not relevant
**PROTOCOL NUMBER / INTERNAL PROTOCOL NUMBER**	APBB1
**HEALTH CONDITION STUDIED**	Oral health
**PHASE**	Not relevant
**OBJECTIVE(S)**	The study objective is to assess the habitual toothbrushing performance and habits in students without relation to dentistry or psychology by means of video filming. Results from video assessment will be related to oral hygiene, several questionnaires on personality traits, body image and previous experiences with dentists and knowledge on oral hygiene.<br>The primary purpose of this study is to explore current relationships and thereby to develop hypotheses for future research on oral health education
**TREATMENT(S)**	None
**INCLUSION CRITERIA**	Willing and able to give written informed consent<br>Student at the University of Freiburg (at least 18 years of age)<br>Not involved in dentistry (e.g. dental nurses or dental students) or in psychology (e.g. students in the field of psychology)<br>Complete (including fixed dental restorations) and closed (except for extraction from orthodontic reasons) dental arches<br>Sufficient German language skills<br>Routine use of manual toothbrush
**EXCLUSION CRITERIA**	Fixed orthodontic appliances<br>Removable dentures<br>Mental or physical disability with the potential to influence oral hygiene<br>Routine use of powered toothbrush

**ENDPOINTS**	First endpoint:<br>correlation between TSI and sub-scores obtained from the questionnaires<br>Other endpoints:<br>Correlation between brushing performance (PI score) and the effective brushing duration, the frequency of alternations between brushing areas and TSI values<br>Analysis of the influence of personal efforts regarding maintenance of oral health (e.g. frequency of visits at the dentist, the frequency of brushing per day) on the brushing performance (PI) und the brushing systematics (TSI)<br>Correlation between the personal efforts regarding maintenance of oral health (e.g. frequency of visits at the dentist, the frequency of brushing per day) and the results of the DKB-35, the Neo-FFI and the PHQ-D.	
**TRIAL DESIGN**	The study is a non-disguised, indirect, and structured observation study with healthy volunteers, no intervention other than the recording, classifying, counting and analysing of data takes place.	
**STATISTICAL ANALYSIS**	For the primary endpoint analysis correlation coefficients for the pairwise correlations between the TSI-score and the single sub-scores obtained from the questionnaires will be computed.<br>The other endpoints will be analysed descriptively using regression models as appropriate for the respective type of data.	
**SAMPLE SIZE**	To be assessed for eligibility	n = 170
	To be allocated/randomised (if applicable) to trial	Not relevant
	To be analysed	n = 170
**TRIAL DURATION**	Recruitment period (months):	Appr. six month
	First patient in to last patient out (months):	Not relevant, no intervention
	Treatment duration per patient (months):	Not relevant, no intervention
	Follow up duration per patient (months):	Not relevant, no intervention
**PLANNED DATES**	Enrolment of first patient, first patient in (FPI)	End of first quarter 2017
	Enrolment of last patient, last patient in (LPI)	End of second quarter 2017
	End of trial defined as last patient last visit (LPLV)	End of second quarter 2017
	Final statistical analysis	End of third quarter 2017
	Planned interim analysis	None

**PARTICIPATING SITES**	Klinik f. Psychosomatische Medizin u. Psychotherapie, Albert-Ludwigs-Universität, Hauptstraße 5, 79104 Freiburg<br><br>Department f. Zahn-, Mund- u. Kieferheilkunde, Klinik f. Zahnerhaltungskunde und Parodontologie, Stiftungsprofessur für Kariesforschung, Albert-Ludwigs-Universität, Hugstetter Straße 55, 79106 Freiburg
**FUNDER(S)**	none

3 ADRESSES AND RESPONSIBILITIES

3.1 ADDRESSES

Study Centres:	Klinik f. Psychosomatische Medizin u. Psychotherapie, Albert-Ludwigs-Universität, Hauptstraße 5, 79104 Freiburg<br><br>Department f. Zahn-, Mund- u. Kieferheilkunde, Klinik f. Zahnerhaltungskunde und Parodontologie, Stiftungsprofessur für Kariesforschung, Albert-Ludwigs-Universität, Hugstetter Straße 55, 79106 Freiburg
Principle Investigators	Prof. Dr. A. Zeeck<br>Tel.: 0761-270-68420<br>e-mail: almut.zeeck@uniklinik-freiburg.de<br>PD Dr. A. Hartmann<br>Tel.: 0761-270-68720<br>e-mail: armin.hartmann@uniklinik-freiburg.de<br>Prof. Dr. N. Schlüter<br>Tel.: 0761-270-48910<br>e-mail: nadine.schlueter@uniklinik-freiburg.de
Investigators	Maxi Müller, Zahnärztin<br>Tel.: 0176-32777960<br>e-mail: maxi.mueller91@web.de<br>Sarah Schorle, cand. med.<br>Tel.: 0157-88387275<br>e-mail: sschorle@arcor.de; sarah.schorle@uniklinik-freiburg.de
Statistician	Kirstin Vach, Dipl.-Math.<br>Tel.: 0761-203-5004<br>e-mail: kv@imbi.uni-freiburg.de<br>Institut für Medizinische Biometrie und Statistik, Universitätsklinikum Freiburg, Hebelstr. 11, Stefan-Meier-Straße 26, 79104 Freiburg

3.2 RESPONSIBILITIES

Principle Investigators

Prof. Schlueter / Prof. Zeeck / PD Dr. Hartmann will be responsible for

- study protocol, CRF, informed consent form, volunteer information form, investigator's file
- study supplies (standard toothbrush)
- volunteer allowance
- submission of study documentation to the relevant ethical committee
- training/calibration of the clinical investigators and quality control
- final clinical report
- questionnaires

Investigators (doctoral students)

Mrs Müller and Mrs Schorle will be responsible for:

- recruitment and inclusion of volunteers
- performance of the clinical phase (plaque index)
- performance of the video filming
- handing out and taking back of questionnaires
- analysis of videos
- documentation of source data and transfer of source data to database

Statistician

Mrs. Vach will be responsible for:

- statistical plan, statistical section of the protocol
- statistical analysis

4 INTRODUCTION

Caries prevention is an established field in dentistry, and due to the variety of public health preventative activities, the caries prevalence is declining in many countries. One main contributing factor is the wide use of topical fluorides via water fluoridation, products for daily home use, or professional applications. There is a tremendous body of literature on fluorides and meanwhile there is strong evidence about the preventive effects of different fluoridation strategies (1). Due to the practicability of fluoridation strategies and it's convincing effects, other fields of caries prevention, dietary counselling and proper tooth brushing, have been much less investigated. There is no doubt that toothbrushing is essential for removal of plaque and debris in order to contribute to good dental and periodontal health (2,3). Proper toothbrushing is of particular importance in preventing gingivitis and periodontitis, and for maintaining the outcome of periodontal treatments.

Thus, oral hygiene strategies have reached scientific interest both in the field of periodontology and in the field of cariology.

The finding that providing oral hygiene advice has only variable success in influencing patient oral hygiene, however, has let to a certain extent of resignation. Most people find it difficult to clean their teeth sufficiently, and the daily experience in dental practice is that patients exhibit plaque even though they reportedly engage in oral hygiene. As a result, oral hygiene instruction is content of preventive programmes, but an under- researched area. Most publications on the topic are from the field of periodontology, but in all there is only little evidence on what brushing technique is most effective or how to influence patient behaviour (4).
For interpreting results from oral hygiene studies, two basic points need to be considered. The first is the theoretical efficacy of tooth cleaning strategies, and it is obvious that the absence of plaque is related to the absence of plaque induced diseases i.e. gingivitis/periodontitis and caries. The second point is the practical efficacy of tooth cleaning strategies, the measure of which in the majority of studies is reduction of plaque levels. The latter, however, is a surrogate parameter as to research questions on oral hygiene techniques and its performance. Lack of reduction of plaque levels does not say anything about the practical efficacy of oral hygiene techniques, as long as nothing is known about the practical performance of oral hygiene, and the skills and dexterity of the included volunteers.

Interestingly, only few studies address these shortcomings of using plaque levels as a surrogate for brushing habits and investigate the brushing performance of volunteers by observing them during practicing the hygiene technique (5–10). These studies, however, have clearly shown that persons, being video-taped during their habitual toothbrushing, mostly brush more or less non-systematically and often do not reach all areas. Often the oral part of the mandible teeth is not brushed (10,11). In addition volunteers alter frequently between predefined areas, probably reducing the brushing efficacy in the respective area. A further very interesting outcome of these studies was that the inter-personally brushing habits show a wide variation; however, intra-personally the brushing habits are very constant or stable (12–14). The latter speaks for a deep rootedness of the movement patterns (15,16).

There are indications that a psychological intervention in combination with oral hygiene instructions can lead to an increase of brushing efficacy; however, the evidence is low (17). Furthermore, nothing is known about the relationship between personality traits (e.g. conscientiousness or neuroticism), body image (e.g. acceptance of one's body) and

brushing performance as well as brushing efficacy. It is quite conceivable that differences in personality traits or experience with dentist as well as dental fear influence the brushing habits. The aim of the present study is therefore to film a group of volunteers (students without relation to any topic of the study – dentistry or psychology) during their habitual oral hygiene and to evaluate the personality traits and body image as well as experience with dentistry by means of different questionnaires.

5 STUDY OBJECTIVE

The study objective is to assess the habitual toothbrushing performance and habits in students without relation to dentistry or psychology by means of video filming. Results from video assessment will be related to oral hygiene, several questionnaires on personality traits, body image and previous experiences with dentists and knowledge on oral hygiene.
The primary purpose of this study is to explore current relationships and thereby to develop hypotheses for future research on oral health education.

6 INVESTIGATIONAL PLAN

6.1 OVERALL STUDY DESIGN AND PLAN

The study is a non-disguised, indirect, and structured observation study with healthy volunteers, no intervention other than the recording, classifying, counting and analysing of data takes place.

6.2 DISCUSSION OF THE STUDY DESIGN

Volunteers in disguised observations tend to act more naturally and the data collected tends to reflect their true reactions. The primary concern with disguised observation however is the ethical concern over recording behavioural information that would normally be private or not voluntarily revealed to a researcher. Therefore, a non-disguised observation design was chosen. To overcome at least partly the disadvantages of a non-disguised observation, the filming was performed through a mirror so that the volunteers should feel less observed than with an obviously visible camera.
Contrary to expectations, however, filming appears to have limited influence on brushing performance. A study comparing video films from volunteers unaware or aware of being filmed revealed that there was no difference in brushing time and influenced performance only to a small extent (16). The assumption that the awareness of being filmed has little effects is also supported by the finding that in repeated video recordings the brushing performance of a volunteer showed a remarkable reproducibility (17). Mierau et al. (18) arrived at similar conclusions when assessing brushing habit patterns in the course of nine

sessions; variations in force, duration, and technique as well as in the sequence of brushing positions and number of changes of brushing sites were small.
Besides limitations due to the observational setting, it must be emphasised that the study population is limited in number and is not representative. Observed results need to be interpreted within these limitations.

6.3 Study centres and technical requirements

The study will be performed at the Department f. Zahn-, Mund- u. Kieferheilkunde, Klinik f. Zahnerhaltungskunde und Parodontologie and at the Klinik f. Psychosomatische Medizin u. Psychotherapie, both located at the Albert-Ludwigs-Universität.

The inclusion of volunteers and the clinical and observational procedures (video recording, plaque indexquestionnaires) and the data management will take place at the Department f. Zahn-, Mund- u. Kieferheilkunde, Klinik f. Zahnerhaltungskunde und Parodontologie. The analysis of the questionnaires at the Klinik f. Psychosomatische Medizin u. Psychotherapie. Data analysis will be primarily done at the Institut für Medizinische Biometrie und Statistik, minor analyses will also be done at the Department f. Zahn-, Mund- u. Kieferheilkunde, Klinik f. Zahnerhaltungskunde und Parodontologie and at the Klinik f. Psychosomatische Medizin u. Psychotherapie.

6.4 Study Population

Volunteers to be included are male and female volunteers living in Freiburg and studying at the University in Freiburg with no relation to dentistry and psychology. They will be recruited via advertisements at noticeboards at central points of the University. Group size is 170.

6.4.1 Inclusion Criteria

- Willing and able to give written informed consent
- Student at the University of Freiburg (at least 18 years of age)
- Not involved in dentistry (e.g. dental nurses or dental students) or in psychology (e.g. students in the field of psychology)
- Complete (including fixed dental restorations) and closed (except for extraction from orthodontic reasons) dental arches
- Sufficient German language skills
- Routine use of manual toothbrush

6.4.2 Exclusion Criteria

- Fixed orthodontic appliances
- Removable dentures
- Mental or physical disability with the potential to influence oral hygiene
- Routine use of powered toothbrush

6.4.3 Blinding

The video films will be analysed under blind conditions as to the questionnaires. This is achieved by retaining the respective parts of the CRF´s until termination of the video assessments. The questionnaires will be analysed under blind conditions as to the video films.

7 FLOW CHART

Study protocol final version and Ethics Commission	
after approval, recruitment of volunteers, volunteers receive appointment, at this appointment:	
	inclusion/exclusion criteria, informed consent, inclusion of the volunteer
	plaque index
	supply of toothbrush
	video recording
	supply of questionnaires
	payment of allowance
after termination of the observation phase:	
	assessment of video recordings
after termination of the assessment of video recordings:	
	transfer from source data to database (questionnaires and raw data of video recording)
	statistical procedures

8 STUDY PARAMETERS

Parameters under study are:

Clinical parameters

Plaque Index (PI) (18). The scores are: 0 – no plaque, 1 – plaque invisible but can be found with periodontal probe at the gingival margin, 2 – moderate plaque easily seen without probing, 3 – ample plaque easily seen. The PI is scored on two surfaces (that is buccal, oral) of all teeth without disclosing. An overall PI score is computed as suggested (18). Additionally, the maximum value per sextant is recorded and the percentage of surfaces showing a PI above 0.

Toothbrushing parameters:

Parameter	Description
Handedness	Hand holding the toothbrush (left, right or both).
Total brushing duration	Time between the first contact of the toothbrush with teeth, and the last action of brushing.
Effective brushing duration	Total brushing duration without interruptions like rinsing, spitting or breaks.
Brushing strokes	Circling, horizontal-linear, vertical-linear, vertical-roll, jiggling (short horizontal) and unspecific brushing movements.
Starting location	The first approached area (oral, vestibular, occlusal, and right side, left side or anterior) at the beginning of toothbrushing.
Brushing events	Frequency of alternations between the sextants, the tooth surfaces (oral, vestibular and occlusal) or a combination of both.
TSI (Toothbrushing Systematics Index)	Analysis and calculation on the basis of the assessed brushing parameters

Questionnaires

- Socio-demographic data (according to Deutsche Mundgesundheitsstudie V (19))
- Toothbrushing education and toothbrushing habits (self-estimation, according to Deutsche Mundgesundheitsstudie V (19))
- Relation to oral hygiene and the own oral cavity
- Experience with dentists
- Dental related self-efficacy (20,21)
- General self-efficacy expectation (Allgemeine Selbstwirksamkeitserwartung (SWE), German version (22))
- Dental Anxiety Scale (original version (23); German version (24))
- NEO-five factors inventory (NEO Fünf-Faktoren-Inventar, NEO FFI (25))
- Dresden Body Image Inventory (Dresdner Körperbildfragebogen, DKB-35 (26))
- Prime MD Patient Health Questionnaire (PHQ, German Version PHQ-D (27), Questions to Depressive Symptoms and to Unspecified Symptoms of Anxiety)

9 PROCEDURES

Volunteers are screened as to the inclusion/exclusion criteria and informed consent is obtained. At the same appointment, the PI is determined, afterwards the volunteers are asked to brush their teeth in their usual way standing/sitting in front of a mirror. Each volunteer is provided with a standard toothbrush. Brushing is performed without toothpaste to facilitate the assessment of the video recordings. While brushing, each volunteer is filmed through the mirror without the investigator present. The video film is saved on the computer with the volunteer’s code as file name. After filming the questionnaires are delivered. After completion of the procedure the allowance is paid.

The video films are analyzed after termination of the observation phase. The data is directly transferred to an excel file, which represent the source data. The brushing duration is determined from the video display; the other parameters are obtained from visual judgment of the volunteer’s behavior. The answers on the questionnaires are transferred to electronic files.

10 QUALITY CONTROL AND DATA MANGEMENT

10.1 GOOD CLINICAL PRACTICE

The study will be conducted along the European directives and ICH Harmonised Tripartite Guideline for good clinical practice E6 (R1 as from June 2017 R2): Note for Guidance on Good Clinical Practice, CPMP/ICH/135/95 Step5.

10.2 Calibration and training of Investigators

The Principal Investigators will perform sufficient calibration and training procedures. Prof. Schlueter will calibrate the clinical investigators as to assessment of PI and to the analysis of the video recordings. Prof. Zeeck will supervise the investigator's analysis of the questionnaires. The Department f. Zahn-, Mund- u. Kieferheilkunde, Klinik f. Zahnerhaltungskunde und Parodontologie will provide sufficient opportunities for practising all procedures and will assure good performance.

10.3 Case Report Forms (CRF)

All of the information collected during the study (PI, questionnaires) will be recorded in the CRF´s identified by the volunteer number. The data obtained from video analysis will be directly transferred to electronic files without noting them in the CRF. An example of the CRF is added in the appendix. The Investigators will ensure that the CRFs are properly and completely filled in. Each page must be signed or initialled by the Investigators, signifying agreement with and responsibility for the recorded data. The Investigators have to identify all data that were directly recorded into the CRF (i.e. no prior written or electronic record of data), and to be considered to be source data.
The CRFs will be checked for completeness and plausibility by the Principal Investigators. The Investigators will resolve any queries.

10.4 Audits and Inspections

The Principle investigators will audit all clinical and assessment procedures in their responsibility on a regular basis. The Investigators and Principle Investigators will make themselves available for each other and will give access to the technical site, the study material, and to volunteer files.
The volunteers' anonymity must be safeguarded and data checked during the audit remain confidential.

10.5 Data Management

All data collected in the CRFs of this study will be entered into a computer database after termination of the video assessments and will be checked for plausibility. Any discrepancies or errors will be clarified and corrected.

11 STATISTICAL METHODS AND DETERMINATION OF SAMPLE SIZE

11.1 STATISTICAL ANALYSIS PLAN

Definition of outcome measures and study objectives

Purpose of the study is to describe quality of current brushing behaviour in adults and thereby to identify needs for further dental education and to associate this with the outcome of the questionnaires to identify predictors for brushing habits.

In a first step the study population will be described with respect to socio-demographic and behavioural characteristics (brushing behaviour). The behavioural characteristics are the following:

- duration of tooth brushing (sec)
- starting location
- type of strokes per sextant
- number of movements between areas
- Toothbrushing systematics

From these data the TSI will be calculated.

In parallel, the information obtained from the questionnaires will be stored in MS-Access-Databases. The scoring of the questionnaires relies on MS-Access-Basic scripts which are already available at the Department of Psychosomatic Medicine and Psychotherapy. The scripts for DKB-35, NEO-FFI and PHQ-D were programmed according to published manuals or articles. These scripts were double checked and debugged. An equivalent procedure will be run for the Dental Anxiety Scale and the Dental Related Self-efficacy. All other forms (e.g. sociodemographic data) do not require standardized scoring procedures. All scores and descriptive data will be exported into a data table, which is compatible with common statistical analysis software (preferably MS-Excel, *.xlsx).

The primary study aim is to correlate the individual brushing habits (effective brushing duration, frequency of alternations between brushing areas, TSI values) with the single scores and sub-scores obtained from the questionnaires.

Secondary objective is to correlate the brushing performance (PI score) with the effective brushing duration, the frequency of alternations between brushing areas and TSI values.

Furthermore the influence of personal efforts regarding maintenance of oral health (e.g. frequency of visits at the dentist, the frequency of brushing per day) on brushing performance (PI) and brushing systematics (TSI) will be analysed.

The third objective is to correlate the personal efforts regarding maintenance of oral health (e.g. frequency of visits at the dentist, the frequency of brushing per day) with the results of the DKB-35, the Neo-FFI and the PHQ-D.

Statistical Analysis

For the primary endpoint analysis correlation coefficients for the pairwise correlations between the TSI-score and the single sub-scores obtained from the questionnaires will be computed.

The other endpoints will be analysed descriptively using regression models as appropriate for the respective type of data.

11.2 SAMPLE SIZE CALCULATION

Calculation of sample size are based on own data from a previous study (5) including a comparable population (students without relation to dentistry). From these data a toothbrushing systematics index was developed (TSI, unpublished results). It is intended to use the index as a measure for differences and changes in systematics of participants. A maximum TSI score of 2 can be reached. Non-instructed participants have a mean index score of 1.2 with a MIN of 0.6, a MAX of 1.6 and a SD of 0.3. A difference in TSI score between systematically and non-systematically habitual toothbrushing of 0.15 can be assumed to be clinically relevant. With $\alpha = 0.05$ and $\beta = 0.1$ a sample size of 170 can be calculated.

12 PROTOCOL AMENDMENTS

Changes or deviations in the conduct of this protocol will be only permitted after discussion with all persons with responsibilities in the study. The Ethics Commission which granted approval for the study must be notified of all changes in the protocol and must provide written approval if changes are substantial (e.g. change of/within study population, number of participants). In the case that an amendment is made, Prof. Schlueter or Prof. Zeeck will be responsible for notification of the Ethics Commission.

13 ETHICS AND REGULATIONS

13.1 ETHICAL CONDUCT OF THE STUDY AND INDEPENDENT ETHICS COMMISSION (IEC) AND RELEVANT AUTHORITIES

This study is to be conducted in accordance with the ethical principles of the Declaration of Helsinki and according to the principle of Good Clinical Practice. Before starting the study,

the study protocol will be subject of review by the IEC. No volunteer should be admitted to the study before the IEC issues its written approval opinion of the study.

13.2 VOLUNTEER INFORMATION AND INFORMED CONSENT

To all volunteers, the Investigator will explain the kind of trial and the risks involved, and the informed consent will be obtained from and signed by each individual volunteer before screening and inclusion of each individual volunteer. The volunteer will also be informed that she/he is free to withdraw from the trial at any time.
Examples of the volunteer information sheet and the informed consent form are given in the appendix.

13.3 VOLUNTEER INSURANCE AND ALLOWANCE

As no invasive intervention is planned no insurance against injury caused by the procedures related to the study is necessary. The allowance will be 15 Euro per volunteer.

13.4 VOLUNTEER DATA PROTECTION AND RETENTION OF STUDY RECORDS

The study will be performed without recording the volunteer’s names, addresses or telephone numbers. All files will be coded by an ID number generated by a random number generator. Other personal data as to social status, age, or gender will be kept under the strict confidence by the Investigator.
The files pertaining to this study will be kept for a period of 10 years from the day of delivery of the final report to the IEC.
A Principal Investigator (Prof. Schlueter) will retain originals of the approved study protocol, copies of completed CRFs, volunteers' participation agreements, relevant source documents and all other supporting documentation related to the study for a period of 10 years. The videos will be backed up on a suitable storage medium. All files must be made available for inspection of all Principal Investigators.

13.5 PUBLICATION / PRESENTATION

The study results will be published, preferably in an indexed journal. Any publication will name either a member of the Department f. Zahn-, Mund- u. Kieferheilkunde, Klinik f. Zahnerhaltungskunde und Parodontologie as first and a member of the Klinik f. Psychosomatische Medizin u. Psychotherapie as last author or vice versa.

References

1. Marinho VCC. Evidence-based effectiveness of topical fluorides. Adv Dent Res. 1. Juli 2008;20(1):3–7.

2. Axelsson P, Lindhe J. Effect of controlled oral hygiene procedures on caries and periodontal disease in adults. J Clin Periodontol. 1978;5:133–51.

3. Axelsson P, Nyström B, Lindhe J. The long-term effect of a plaque control program on tooth mortality, caries and periodontal disease in adults. Results after 30 years of maintenance. J Clin Periodontol. September 2004;31(9):749–57.

4. Clarkson JE, Young L, Ramsay CR, Bonner BC, Bonetti D. How to influence patient oral hygiene behavior effectively. J Dent Res. Oktober 2009;88(10):933–7.

5. Schlueter N, Klimek J, Saleschke G, Ganss C. Adoption of a toothbrushing technique: a controlled, randomised clinical trial. Clin Oral Invest. 2010;14:99–106.

6. Schlueter N, Klimek J, Ganss C. Relationship between plaque score and video-monitored brushing performance after repeated instruction--a controlled, randomised clinical trial. Clin Oral Investig. März 2013;17:659–67.

7. Rugg-Gunn AJ, Macgregor ID, Edgar WM, Ferguson MW. Toothbrushing behaviour in relation to plaque and gingivitis in adolescent schoolchildren. J Periodontal Res. 1979;14:231–8.

8. Rugg-Gunn AJ, Macgregor ID. A survey of toothbrushing behaviour in children and young adults. J Periodontal Res. 1978;13:382–8.

9. Macgregor ID, Rugg-Gunn AJ, Gordon PH. Plaque levels in relation to the number of toothbrushing strokes in uninstructed English schoolchildren. J Periodontal Res. 1986;21:577–82.

10. Winterfeld T. Toothbrushing and flossing habits in young adults : a video-based observational study [Internet]. [Giessen]: Justus-Liebig-University; [zitiert 2. September 2016]. Verfügbar unter: http://geb.uni-giessen.de/geb/volltexte/2015/11634/

11. Winterfeld T, Schlueter N, Harnacke D, Illig J, Margraf-Stiksrud J, Deinzer R, u. a. Toothbrushing and flossing behaviour in young adults-a video observation. Clin Oral Investig. 2015;19:851–8.

12. Winterfeld T, Schlueter N, Winterfeld K, Ganss C. Effect of systematic toothbrushing on changes of toothbrushing behaviour and relation to plaque (Abstr.). Caries Res. 2014;48:412–3.

13. Winterfeld T, Schlueter N, Klimek J, Ganss C. Consistency of Toothbrushing Habits: A Pilot Video Analysis Study; Abstract: 60th ORCA Congress. Caries Res. 2013;47:500.

14. Ganss C, Schlueter N, Preiss S, Klimek J. Tooth brushing habits in uninstructed adults-frequency, technique, duration and force. Clin Oral Invest. 2009;13:203–8.

15. Shiffrin RM, Schneider W. Controlled and automatic human information processing: II. Perceptual learning, automatic attending, and general theory. Psychol Rev. 1977;84:127–90.

16. Schneider W, Shiffrin RM. Controlled and automatic human information processing: I. Detection, search, and attention. Psychol Rev. 1977;84:1–66.

17. Renz A, Ide M, Newton T, Robinson PG, Smith D. Psychological interventions to improve adherence to oral hygiene instructions in adults with periodontal diseases. CochraneDatabaseSystRev. 2007;CD005097-.

18. Silness J, Loe H. Periodontal disease in pregnancy. II. Correlatation between oral hygiene and periodontal condition. Acta Odontol Scand. Februar 1964;22:121–35.

19. Jordan R, Micheelis W. Fünfte Deutsche Mundgesundheitsstudie DMS V. Deutscher Ärzte-Verlag; 2016. (Materialienreihe; Bd. 35).

20. Syrjälä AMH, Knuuttila MLE, Syrjälä LK. Self-efficacy perceptions in oral health behavior. Acta Odontol Scand. 2001;59:1–6.

21. Syrjälä AM, Kneckt MC, Knuuttila ML. Dental self-efficacy as a determinant to oral health behaviour, oral hygiene and HbA1c level among diabetic patients. J Clin Periodontol. September 1999;26(9):616–21.

22. Schwarzer R, Jerusalem M. Skalen zur Erfassung von Lehrer- und Schülermerkmalen - Dokumentation der psychometrischen Verfahren im Rahmen der Wissenschaftlichen Begleitung des Modellversuchs Selbstwirksame Schulen. Berlin; 1999 S.

23. Corah NL. Development of a dental anxiety scale. J Dent Res. August 1969;48(4):596.

24. Margraf-Stiksrud J. Dental Anxiety Scale (DAS). In: Angstdiagnostik. Berlin: Springer; 2003. S. 415–8.

25. Costa PT, McCrae RR. Normal personality assessment in clinical practice: The NEO Personality Inventory. Psych Assess. 1992;4(1):5–13.

26. Pöhlmann K, Roth M, Brähler E, Joraschky P. [The Dresden Body Image Inventory (DKB-35): validity in a clinical sample]. Psychother Psychosom Med Psychol. März 2014;64(3–4):93–100.

27. Löwe B, Spitzer RL, Zipfel S, Herzog W. Gesundheitsfragebogen für Patienten (PHQ D). Komplettversion und Kurzform. Testmappe mit Manual, Fragebögen, Schablonen. 2. Aufl. Karlsruhe: Pfizer; 2002.
