# Supplementary material for: Relationship between dental experiences, oral hygiene education and self-reported oral hygiene behaviour
Source: PLoS One. 2022 Feb 24;17(2):e0264306. doi: 10.1371/journal.pone.0264306 (PMC8870456; doi:10.1371/journal.pone.0264306)
Supplement: Attachment — Submitted filename: Response to Reviewers.docx [file pone.0264306.s009.docx]

**Response to Reviewers**

*Dear Dr. Heber, dear reviewers,*

*Thank you very much for reviewing our manuscript again and for your constructive feedback. We have revised the manuscript in light of the reviewers' comments. The changes are marked in yellow in the manuscript. Please see below for a point-by-point response to the reviewers comments.*

**Reviewer #1:** Minor comments

The accepted level of significant for p value to be stated.

*The accepted level of significant for p value is now stated in the section „Materials and Methods – Procedures (Data handling, questionnaire evaluation and statistical analysis) (Line 262-263)*.

There was no data presented in table form for S1 Table (S1 Table consists only questions)

*We have added the results of the questionnaires of the S1 table in tabular form to the "supporting information" under a new S2 Table; the other Supporting Information numbers have been changed accordingly.*

The figures in the text (results section) should refer to/cite S4 Table and not S2 Table as there were no figures presented in S2 Table. As such S2 Table to be replaced with S4 Table in the text and followed by respective domains and items.

*The references in the results and discussion section have been changed to the respective domain and item numbers as presented in S5 Table (previously S4).*

**Reviewer #3:** the changes that has been done considering the english in the abstact and introduction is accurate.

*Thank you for the appreciative comment.*

**Reviewer #4:** The authors have presented the manuscript well and all comments have been addressed appropriately. The data given is supporting the conclusion.

*Thank you for the appreciative comment.*
